# Changes in glucose metabolism after distal pancreatectomy: a nationwide database study

**DOI:** 10.18632/oncotarget.24325

**Published:** 2018-01-27

**Authors:** Jin-Ming Wu, Te-Wei Ho, Ching-Yao Yang, Po-Huang Lee, Yu-Wen Tien

**Affiliations:** ^1^ Department of Surgery, National Taiwan University Hospital and National Taiwan University College of Medicine, Taipei, Taiwan, ROC

**Keywords:** distal pancreatectomy, chronic pancreatitis, dyslipidemia, glucose metabolism, nationwide database

## Abstract

**Background:**

This population-based study evaluated changes in glucose metabolism after distal pancreatectomy (DP).

**Methods:**

Data from the Taiwan National Health Insurance Research Database was collected from 2001 to 2010. Of 1,980 patients who underwent DP, 507 had diabetes and 1,410 did not.

**Results:**

Of the 1,410 non-diabetic pre-DP patients, 312 (22.1%) developed newly-diagnosed diabetes after DP. Multiple logistic regression analysis revealed that dyslipidemia [hazard ratio = 1.940; 95% confidence interval = 1.362–2.763; *P* < 0.001] and chronic pancreatitis (hazard ratio = 2.428; 95% confidence interval = 1.889–3.121; *P* < 0.001) were significantly associated with the development of diabetes after DP. On the other hand, analysis of changes in glucose metabolism among 289 pre-DP diabetes without the use of insulin revealed that 173 (59.9%) had deteriorated glucose metabolism after DP.

**Conclusion:**

Dyslipidemia and chronic pancreatitis are risk factors for the development of diabetes. Further, more than half of the pre-DP diabetes patients without the use of insulin had deterioration of glucose metabolism after DP. Therefore, clinicians should monitor glucose metabolism and clinical symptoms of hyperglycemia among DP patients.

## INTRODUCTION

Distal pancreatectomy (DP) is the standard surgical procedure for resection of neoplastic and non-neoplastic lesions of the pancreatic body and tail [1]. The number of DP procedures has recently increased because of improvements in the detection of cystic and borderline pancreatic neoplasms (most develop in the distal pancreas) [2, 3], and increase in the indications of pancreatic surgery for benign or low-grade malignant tumors [4]. In addition, DP can provide relief from pain associated with chronic pancreatitis (CP) in the left pancreas and thus improve quality of life [5].

The pancreas plays a major role in glucose metabolism via endocrine hormones and secretes various digestive juices into the duodenum as an exocrine gland [6]. As such, DP may result in deterioration of glucose homeostasis and exocrine pancreatic insufficiency (EPI) because of the loss of the pancreatic parenchyma. Pancreatectomy-associated diabetes mellitus (DM) is defined as the onset of DM after pancreatectomy (pancreatogenic DM). The American Diabetes Association classifies this type of DM as “other specific type of diabetes mellitus” [7]. The rates of pancreatogenic (type 3c) DM vary from 9% to 39%, depending on the extent of DP as well as the underlying disease [1, 8]. Moreover, associated EPI after pancreatectomy may result in the deterioration of nutritional status, which in turn deteriorates glucose metabolism [9].

The incidence of pancreatic endocrine insufficiency after DP may increase because the follow-up period after surgery becomes longer, and more DPs are being performed for non-malignant diseases in younger patients who have long life expectancy. In addition, DP performed for CP may be associated with a higher incidence of pancreatic insufficiency due to the loss of pancreatic tissues and previous/ongoing damage to the residual pancreatic parenchyma [10]. However, some reports have indicated that many patients have normal fasting postoperative blood glucose levels after DP and there is little effect on pancreatic exocrine function [5, 11]. Furthermore, the pre-DP DM subjects may experience deterioration of glucose metabolism after DP because of the loss of pancreatic parenchyma. To date, few studies have investigated the incidence of post-DP pancreatogenic DM after resection of benign or malignant pancreatic diseases with a long follow-up period.

The purpose of this study was to retrospectively examine the long-term changes in pancreatic endocrine function after DP using data from the Taiwan National Health Insurance Research Database (NHIRD).

## RESULTS

### Incidence of endocrine pancreatic insufficiency in non-DM subjects after DP

**Figure 1:**
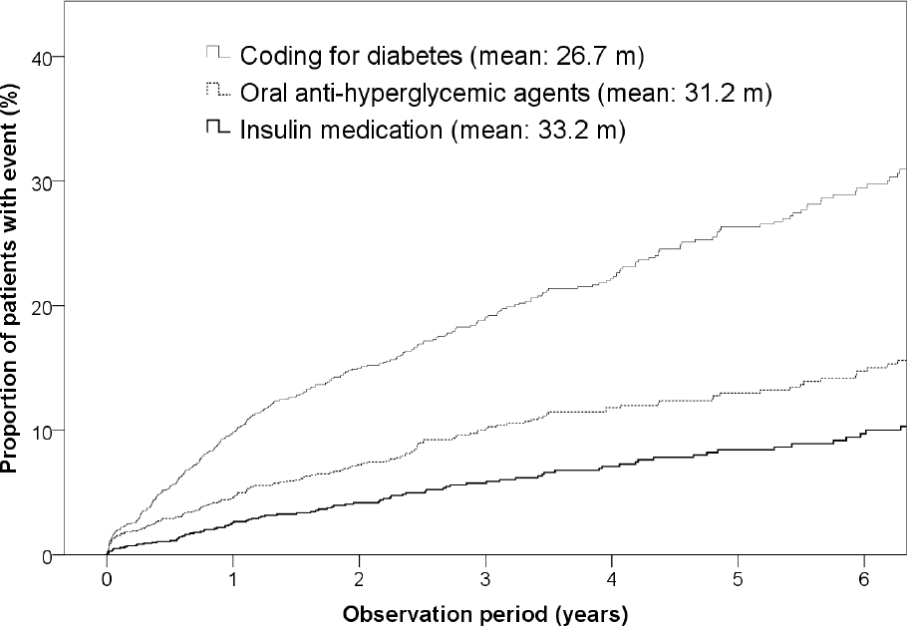
Proportion of patients with endocrine insufficiency requiring OHAs and insulin in pre-DP non-DM subjects by time after DP

Among the 1,410 patients who underwent DP, 312 (22.1%) were newly diagnosed with pancreatic endocrine insufficiency postoperatively. In this study, there were 65.2 per 1000 person-years of newly-diagnosed diabetes in patients with DP, which is considerably higher than that in the general Taiwanese population (with an incidence of 7.6 per 1000 person-years) [12, 13]. The results of the univariate comparisons of patients with and without pancreatic endocrine insufficiency after DP are shown in Table 1. Patients who developed DM after DP were more likely to be male (*P* = 0.047), have dyslipidemia (*P* < 0.001), and have CP (*P* < 0.001). Cox logistic regression analysis showed that dyslipidemia (HR = 1.940; 95% CI = 1.362–2.763; *P* < 0.001) and CP (HR = 2.428; 95% CI = 1.889–3.121; *P* < 0.001) were significantly associated with pancreatic endocrine insufficiency after DP (Table 2). Further, proportion of patients with endocrine insufficiency requiring OHAs and insulin in pre-DP non-DM subjects by time after DP was shown in Figure 1.

**Table 1:** Univariate analysis of the influence of demographic and clinical characteristics on endocrine pancreatic insufficiency after distal pancreatectomy

	Total (*N* = 1410)	Non-DM (*n* = 1,098)	DM (*n* = 312)	*P* value
Female	677 (48)	543 (50)	134 (43)	.047
Age group (y)				.337
≤49	685 (49)	543 (50)	142 (46)	
50–64	390 (28)	294 (27)	96 (31)	
≥65	335 (24)	261 (24)	74 (24)	
Age (y)	51.5 ± 16.3	51.0 ± 16.6	52.9 ± 15.2	.056
Concomitant splenectomy	266 (19)	208 (19)	58 (19)	.935
Charlson comorbidity index				.459
0–1	486 (35)	373 (34)	113 (36)	
≥2	924 (66)	725 (66)	199 (64)	
Comorbidity				
Peptic ulcer disease	729 (52)	552 (50)	177 (57)	.057
Hypertension	390 (28)	293 (27)	97 (31)	.062
Dyslipidemia	95 (7)	59 (5)	36 (12)	<.001
Chronic pancreatitis	245 (17)	142 (13)	103 (33)	<.001
Renal failure	32 (2)	21 (2)	11 (4)	.128
Indication for DP				.393
Pancreatic cancer	186 (13)	140 (13)	46 (15)	
Other causes	1224 (87)	958 (87)	266 (85)	
Monthly income (NT$)				.080
≤22,798	806 (57)	614 (56)	192 (62)	
>22,798	604 (43)	484 (44)	120 (39)	
Duration of follow-up (months)				
Median (IQR)	37.8 (18.7–73.0)	33.8 (17.3–66.7)	59.2 (25.9–96.2)	<.001
Mean, log-rank	48.7 ± 35.4	44.7 ± 33.4	63.0 ± 38.4	<.001

DM, diabetes mellitus; IQR, interquartile range.

Age data are presented as the mean ± standard deviation and other data as the number (percentage).

**Table 2:** Multivariate analysis of the influence of demographic and clinical characteristics on endocrine pancreatic insufficiency after distal pancreatectomy

	Estimate	SE	Wald	HR	95% CI	*P* value
Female	−0.209	0.117	3.161	0.812	0.645–1.022	.075
Dyslipidemia	0.663	0.180	13.485	1.940	1.362–2.763	<.001
Chronic pancreatitis	0.887	0.128	47.909	2.428	1.889–3.121	<.001

CI, confidence interval; DM, diabetes mellitus; HR, hazard ratio; SE, standard error, Wald, Wald chi-square.

### Deterioration of glucose metabolism in DM subjects without insulin use

**Figure 2:**
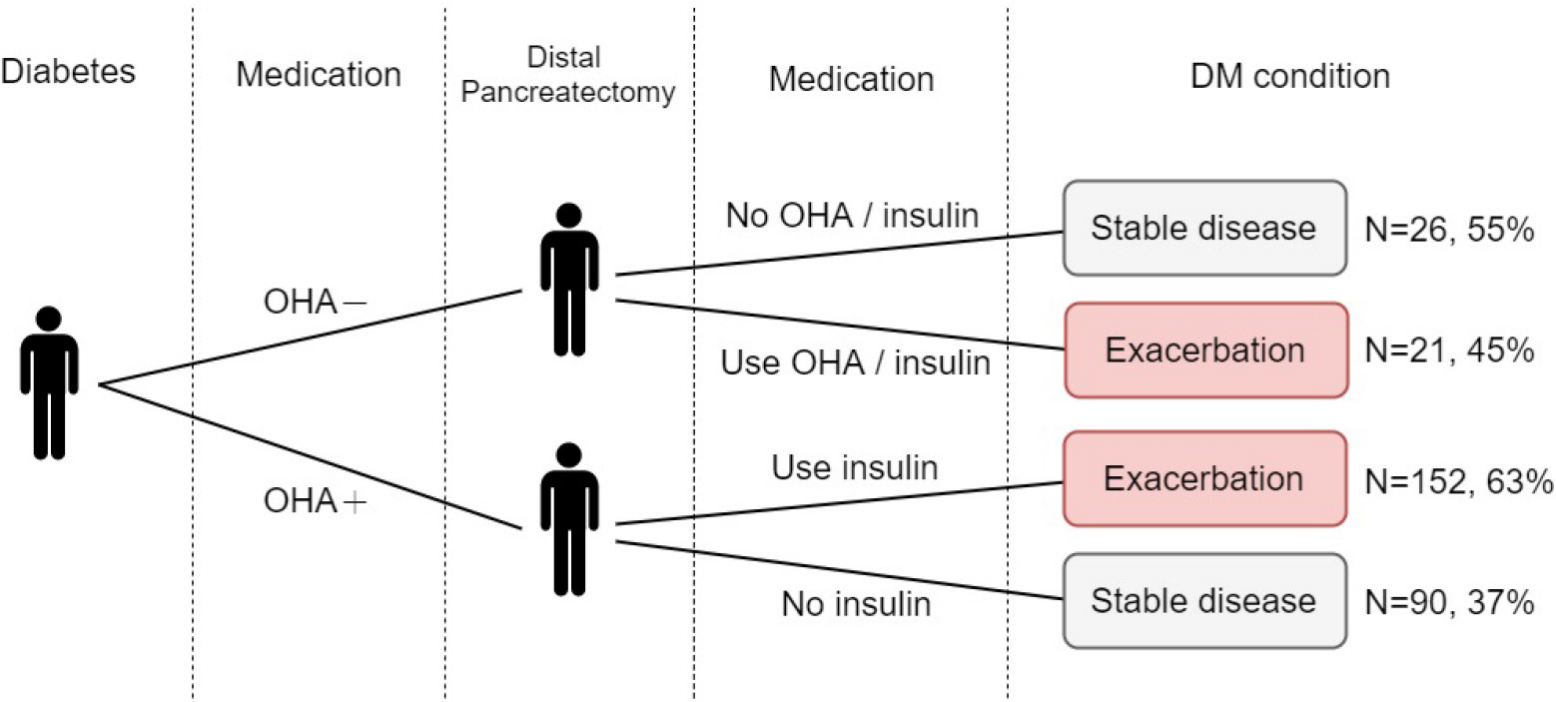
Deterioration of glucose metabolism in pre-distal pancreatectomy diabetes mellitus cases without the use of insulin

Of the 289 patients with pre-DP DM without the use of insulin, 47 were treated with an AHM and 242 with OHAs. Overall, deterioration of glucose metabolism after DP was observed in 173 patients (59.9%), including 21 (45%) who received no AHM and 152 (63%) who were treated with OHAs (Figure 2).

## DISCUSSION

This study found that 22.1% of patients who underwent DP developed DM. CP and dyslipidemia are two significant factors contributing to post-DP pancreatic endocrine insufficiency. CP is a disease characterized by progressive pancreatic inflammation and fibrotic injury, causing irreversible parenchymal damage and resulting in loss of endocrine and exocrine function [14]. Furthermore, pancreatic resection for CP further deteriorates glucose metabolism, which is clearly associated with the extent of parenchyma resection and also with the progression of underlying pancreatitis [15]. Compared to DP for CP, resection of pancreatic tumors apparently is associated with a lower incidence of endocrine dysfunction. The rate of new-onset DM after DP in patients without pancreatitis is relatively low, at approximately 5%–9% [1, 11, 16]. For patients with CP, the reported risk of DM after DP is around 22%–50%, which is similar to our findings [5, 17–19].

In addition, the results of this study showed that dyslipidemia is a risk factor for post-DP DM. Dyslipidemia is a risk factor for the development of nonalcoholic fatty pancreas disease, which may reduce β-cell function, probably via lipotoxicity [20]. Some clinical studies support the association between a fatty pancreas and an increased risk of DM [21, 22]. In contrast, other studies, in which computed tomography was used to assess the severity of fatty pancreas, do not support the association between a fatty pancreas and DM [23, 24]. These inconsistent findings may be related to the definition of a fatty pancreas based on the imaging method, race, duration of follow-up, and associated metabolic syndromes [25].

Furthermore, changes in glucose metabolism of pre-DP DM without the use of insulin were analyzed, which showed deterioration of glucose metabolism in 59.9% of patients after DP. Although such cases were anticipated to have relative dysfunction of glucose metabolism before DP, the deterioration of glucose metabolism was clinically significant afterward, thus clinicians should inform patients of this information, closely monitor blood sugar parameters, and appropriately adjust anti-hyperglycemic strategies.

In our report, some demographics are different from other studies, and the reasons are illustrated below. First, the mean age is 51.5 years, which is a relatively young population. The mean age of the patients with distal pancreatectomy (DP) in the National Surgical Quality Improvement Project (USA) and in the Surveillance Epidemiology and End Results was 61.6 and 63.4 years, respectively [26]. According to one Taiwan population study, more than 60% of patients with newly-diagnosed diabetes were aged >60 years [12]. To investigate changes in glucose metabolism, we only enrolled individuals without diabetes. Because of this, our cohort was likely younger than other population studies that focused on a broader sample of patients with DP. Second, half of patients had peptic ulcer disease in this cohort. One study determined that gastric ulcers increased the risk of pancreatic cancer during an 18-year follow-up period [27]. In addition, most patients who undergo DP also receive a gastroduodenoscopic examination or endoscopic ultrasound to do biopsy of the pancreatic tumor or to gain information regarding the character of the tumor. Occult cases of PUD may be diagnosed via endoscopic examination. Furthermore, some cases of DP were associated with multiple endocrine neoplasm syndromes, which may result in peptic ulcer diseases, particularly in cases with gastrinoma. We believe these are the reasons for the high observed incidence of peptic ulcer disease in this study cohort. Third, only 7% population had dyslipidemia. Obesity is not a common disorder in Taiwan; therefore, dyslipidemia (a metabolic syndrome) is also not as common as it is in Western countries. Approximately 15%–30% of Taiwanese individuals are diagnosed with dyslipidemia, depending on age and gender. Moreover, the subjects with pancreatic neoplasms may have cancer cachexia, which causes body weight loss and nutritional status deterioration. Further, our study population included only subjects without diabetes, who had a relatively low incidence of dyslipidemia compared to patients with diabetes. These reasons may explain the low incidence of dyslipidemia in this study cohort.

With the advancement of computational and technical methods in metabolome, both amino acid/ fatty acid derivative metabolites were approved to associated with pathogenesis of diabetes and related risk factors [28]. Increases in plasma levels of branched-chain and aromatic amino acids have been considered not only as a marker of insulin resistance but also as a predictive risk for development of type 2 diabetes [29, 30]. Conversely, the plasma levels of glycine and glutamine were decreased in patients with insulin resistance [31, 32]. Among the lipid metabolome, long-chain fatty acids and short & odd-chain acylcarnitines contributed to pathophysiological impairment of glucose metabolism [33, 34]. From a clinical point of view, further studies will be required to fully integrate these findings into discovery of the diabetes pathophysiology on DP patients.

One of the strengths of this study lies in its use of a nationwide population-based approach to characterize long-term changes in endocrine insufficiency after DP from high-quality databases [35–37]. However, this study also had some limitations. First, the analyzed data did not include body weight, family history, laboratory examination [28] results, or risk factors for the development of DM, such as alcohol use and other lifestyle factors. Second, coding errors may have existed in the administrative database. To decrease the incidence of coding errors, the diagnostic accuracy of comorbidities was confirmed if patients had one relevant inpatient or three outpatient ICD-9 codes separated by at least 30 days. Further, strict criteria (*P* < 0.01) were used to evaluate the significance of associations between independent variables and the occurrence of endocrine insufficiency. Third, we agree that some key metabolite changes (levels of lactate, fatty acids, and amino acids) are very important factors in glucose metabolism. However, these data were unavailable in the nationwide administrator database. Nationwide population-based cohort studies are crucial for determining risk factor-outcome associations. We examined subjects with DP with extended follow-up periods to investigate changes in glucose metabolism after DP. Although we identified some risk factors in this study, the mechanisms that connect risk factors and diabetes were assumed from a previous hypothesis, which should be elucidated in experimental animal models. Fourth, volume of pancreas tissue resected was a very important factor associated with development of diabetes. Pancreatic beta cells produce insulin, which is essential for glucose hemostasis [38]. Autopsy research indicated that resection of more than 65% of beta cells can cause development of overt diabetes [39]. Two observational studies used computed tomography to assess loss of pancreatic parenchyma. This study found that resection of more than 44% of pancreatic volume was associated with new-onset diabetes [40, 41]. Because of shortcomings of the nationwide database, data pertaining to loss of pancreatic tissue due to DP were not available. However, beta cells are also associated with other diseases (such as dyslipidemia, alcohol use, or pancreatitis), which can contribute to beta cell deterioration, in addition to pancreatic volume loss. On the other hand, exocrine pancreatic insufficiency (EPI) may develop after DP because the pancreas is an exocrine organ involved in food digestion. In the present study, exocrine insufficiency was defined as the use of exogenous pancreatic enzymes for more than three consecutive months [18, 42], and 615 (43.6%) of the 1,410 non-diabetic patients developed new EPI after DP. Of the 312 patients with newly-diagnosed diabetes, 176 (56.4%) also developed post-DP EPI. We realized that pancreatic resection volume is a crucial factor related to the development of DM as well as EPI. However, the volumetric cut-off point for pancreatic resection in EPI may be different from that in DM. In addition, the distribution of pancreatic exocrine tissues throughout the whole pancreas may be unique to pancreatic endocrine tissues. These assist in discrimination of DM from EPI in patients with DP.

In summary, patients with dyslipidemia and CP had higher rates of newly-diagnosed pancreatic endocrine insufficiency among non-diabetic patients in a nationwide DP cohort. Further, more than half of the pre-DP DM patients without the use of insulin had deterioration of glucose metabolism after DP. Hence, clinicians should monitor glucose metabolism and clinical symptoms of hyperglycemia among DP patients.

## METHODS

The protocol of this retrospective study was approved by the Institutional Review Board of National Taiwan University Hospital (approval no. 201606084W). The requirement to obtain informed consent was waived because sensitive information (patient identification data, medical institutions, and medical staff) was encrypted to protect privacy.

### Data source

Taiwan launched a mandatory national health insurance program to offer comprehensive medical care coverage to all citizens in 1995. The entire NHIRD was released for research purposes by the Collaboration Center of Health Information Application, Ministry of Health and Welfare in 2014. The NHIRD contains original claim data of more than 23 million people, which is 99.9% of the entire population of Taiwan [43]. Hence, the database provides a large and valuable population-based source for epidemiological research. The NHIRD includes all information on outpatient and inpatient claims, patient demographic information, and related information pertaining to prescriptions, procedures, and medications. All clinical diagnoses and procedures were recorded according to the International Classification of Diseases, Ninth Revision, Clinical Modification (ICD-9-CM) coding scheme.

### Study cohort

**Figure 3:**
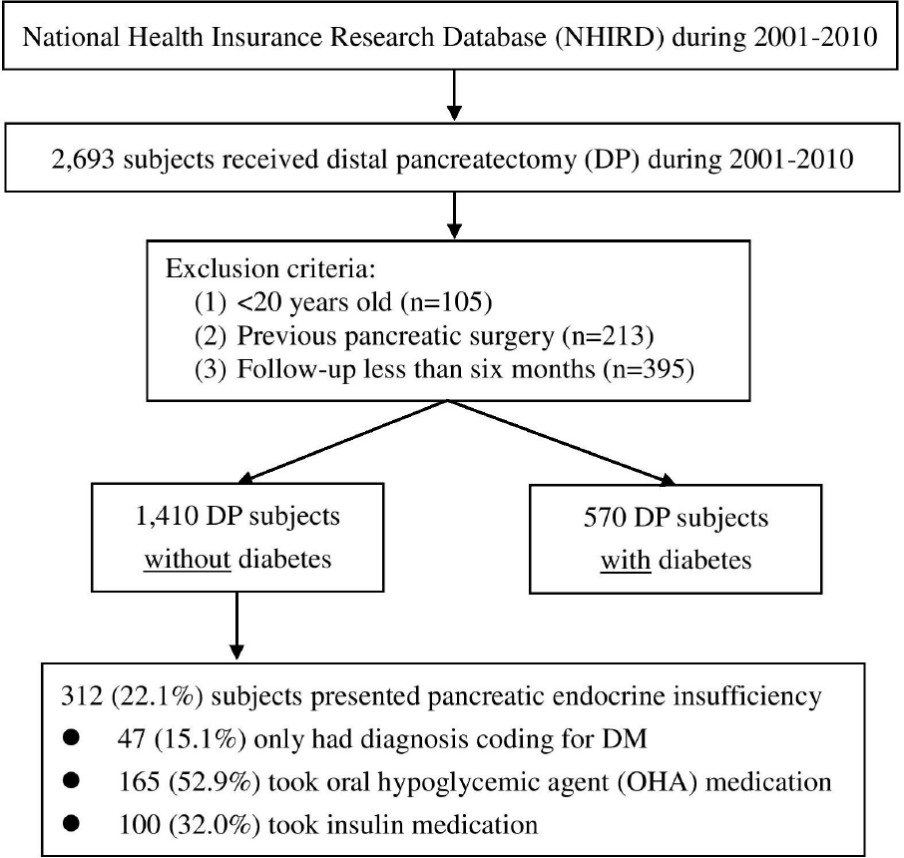
Study flow diagram

A flow diagram of patient inclusion for this study is shown in Figure 3. The study cohort consisted of all patients who underwent DP (ICD-9 procedure code 52.52) between 2001 and 2010 (*N* = 2,693). The exclusion criteria for the current analysis were: (1) age >20 years (*n* = 105); (2) previous pancreatic surgery (*n* = 213); and (3) follow-up of less than 6 months (*n* = 395). Thus, the final sample of this study was comprised of 1,410 non-DM and 570 DM patients who underwent DP between 2001 and 2010. Of the 570 DM subjects, 281 received insulin to control hyperglycemia. The above cases were excluded because of pre-DP poor reserve of pancreatic endocrine function.

### Outcome measures

The outcomes of this study were pancreatic endocrine insufficiency of pre-DP non-DM cases and deterioration of glucose metabolism of pre-DP DM cases without the use of insulin. Endocrine insufficiency was defined as a new diagnosis of DM after DP. For the purposes of this study, DM was defined as two or more outpatient clinic visits or one admission with a diagnostic code of DM (ICD-9-CM code 250) [42]. This definition of DM was validated in a previous NHIRD study in which the sensitivity was 93.2% and the positive predictive value was 92.3% [44]. The use of oral anti-hyperglycemic agents (OHAs) and insulin was also examined from the pharmacy prescription database. Deterioration of glucose metabolism was defined as either pre-DP DM treated with an oral hypoglycemic medication, but without the use of an anti-hyperglycemic medication (AHM; OHAs or insulin) or pre-DP DM treated with OHAs and insulin after DP.

### Covariates

Comorbidity data of patients prior to DP were also collected. Comorbidities were identified by the following ICD-9-CM codes: concomitant splenectomy during DP (41.5), peptic ulcer disease (531–535), dyslipidemia (272.0–272.2), CP (577.1), renal failure (584.x–586.x), hypertension (401.x–405.x), and pancreatic cancer (157.0–157.9). The diagnostic accuracy of cancer was confirmed by the Registry for Catastrophic Illness Patient Database, a subpart of the NHIRD [42]. The Charlson comorbidity index was calculated to assess the severity of comorbidities for each patient [45]. The demographic characteristics investigated were sex, age (≤49, 50−64, and ≥65 years), and monthly income (New Taiwan dollar ≤22,798 or >22,798).

### Statistical analysis

Microsoft SQL Server 2008 was used for the management of the NHIRD and baseline data. Data analysis was performed using IBM SPSS statistical software v20 (IBM Corp., Armonk, NY, USA). All variables were calculated as percentages and quantitative variables are summarized as the mean ± standard deviation. The Student’s *t*-test was used for analysis of continuous variables. Nominal variables were analyzed using the chi-square test and Fisher exact test if the cell count was <5. To evaluate the independent effects of factors on endocrine insufficiency or deterioration of glucose metabolism after DP, predictors were examined with Cox regression analysis and the results are presented as hazard ratios (HRs) with 95% confidence intervals (CIs). Next, the best candidate covariates (*P* value < 0.05) were entered into a multivariate model. A two-sided *P* value of < 0.01 was considered statistically significant in the multivariate model.

## Abbreviations

DP: Distal pancreatectomy
CI: confidence interval
DM: diabetes mellitus
NHIRD: National Health Insurance; Research Database
ICD-9-CM: International Classification of Disease, Ninth Revision, Clinical Modification
CCI: Charlson Comorbidity Index
